# The Avon Longitudinal Study of Parents and Children (ALSPAC): a 2022 update on the enrolled sample of mothers and the associated baseline data

**DOI:** 10.12688/wellcomeopenres.18564.2

**Published:** 2023-09-06

**Authors:** Daniel Major-Smith, Jon Heron, Abigail Fraser, Deborah A. Lawlor, Jean Golding, Kate Northstone

**Affiliations:** 1 Population Health Sciences, University of Bristol, Bristol, BS8 2BN, UK; 2 MRC Integrative Epidemiology Unit, University of Bristol, Bristol, UK; 3 NIHR Biomedical Research Centre, University of Bristol, Bristol, UK

**Keywords:** ALSPAC, Children of the 90s, Birth cohort study, Cohort profile, Enrolment

## Abstract

The Avon Longitudinal Study of Parents and Children (ALSPAC) is a prospective birth cohort, based in and around Bristol, UK, established to explore genetic and environmental factors impacting health and development. 14,541 pregnancies were initially recruited from 20,248 eligible pregnancies. As the G1 (Generation-1) offspring turned 7 years of age, children from eligible pregnancies who had not been enrolled into the study were invited to take part. The enrolment status of these additional G1 offspring (n=913) has been well-documented. Here we provide an updated description of the ALSPAC G0 (Generation-0) mothers study cohort (which includes newly enrolled mothers), their associated pregnancies and the *mz* data file that defines this cohort. At the time of writing there are 14,833 unique mothers enrolled in ALSPAC, with 15,447 associated pregnancies enrolled. The update to the *mz* file also includes new variables to assist researchers when using mothers’ data whilst accounting for non-independence between data related to multiple pregnancies (i.e., women with more than one pregnancy in the study).

## Introduction

The Avon Longitudinal Study of Parents and Children (ALSPAC; known locally as ‘Children of the 90s’) is a longitudinal birth cohort. It recruited pregnant women with expected delivery dates between April 1991 and December 1992 who were resident in that part of the former county of Avon contained within the South-West of England Regional Health Authority ^1, 2^. 14,541 pregnancies were enrolled (known as the ‘core’ ALSPAC sample). The subsequent children, their parents and families have been followed up ever since, via questionnaires, hands-on clinic assessments, and linkage to routine data. ALSPAC is currently a three-generation cohort study, which includes: the mothers and their partners, (Generation-0; ALSPAC-G0); the original study children (Generation-1; ALSPAC-G1) and; the offspring of the original study children (Generation-2; ALSPAC-G2 ^3^). The study website contains details of all the data that is available through a searchable data dictionary.

When the G1 children were around 7 years of age, attempts were made to bolster the initial sample with eligible cases who had not joined the study originally. From that point onwards any eligible participants (G0 and G1) have been welcomed into the study. These recruitment phases have been well-documented for the G1 cohort ^2, 4^. However, the impact of this additional recruitment phase on the G0 mother's cohort has not been described in detail.

The aim of this data note is to describe new G0 mothers' variables that have been made available (in the *mz* file) and to provide an up-to-date description of the cohort, including non-core G0 mother recruitment until September 2021 (the start of the ALSPAC @30 clinic; i.e., follow-up of the G1 offspring at age 30, in addition to the G0 mothers and their partners) as well as up to date mortality data. We highlight that the ALSPAC-G0 cohort includes some mothers who have had two pregnancies (including early miscarriages). While this has been acknowledged previously ^1^, we describe new variables that allow researchers to identify these women, their pregnancies/offspring, and to account for the non-independence between data points. We also provide practical guidance on potential approaches to do this.

## Methods

The Project to Enhance ALSPAC through Record Linkage (PEARL) retrospectively defined the full eligible G1 cohort through linkage to NHS delivery records and child health records ^2^. As ALSPAC is a pregnancy-based cohort, this campaign defined the eligible G0 mother’s cohort at the same time. After core enrolment during pregnancy, additional phases of G1 enrolment occurred at the age of 7, between 8 and 18 and between 18 and 26 years of age ^4^. These new cases have had a G1 focus and the recruitment of additional G0 mothers has not been defined. Here we will define G0 mothers as either ‘core’ enrolees (i.e., enrolled during pregnancy), or ‘non-core’ enrolees (enrolled from when their children were aged 7 years onwards). It should be noted that PEARL could not identify all pregnancies that ended in miscarriage and did not present to prenatal care and so the eligible cohort may have been under-estimated.

## Study numbers

### G0 mothers’ recruitment

**Figure 1.**
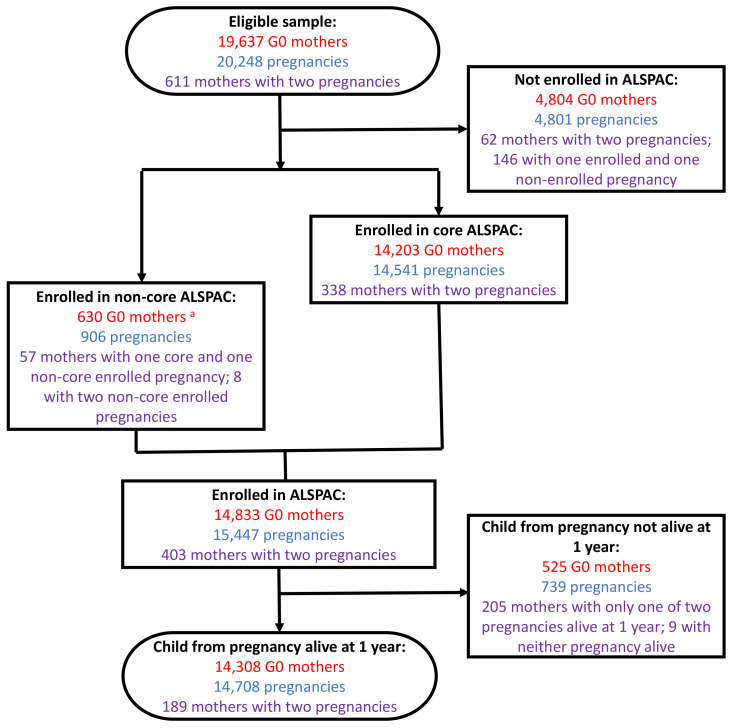
The ALSPAC-G0 mother’s enrolment flow diagram. At each phase of enrolment, the figure shows the number of G0 mothers (red), the number of associated pregnancies (blue), and the number of G0 mothers with two pregnancies in ALSPAC (purple). ^a^ Note that there were 211 non-core G1 enrolments not associated with a G0 mother enrolment (in which case the pregnancy is enrolled but the G0 mother is not). 57 G0 mothers with both a core enrolled and non-core enrolled pregnancy are also excluded from this total (as they are counted in the ‘core ALSPAC’ mothers total).

A flow diagram of recruitment of G0 mothers is presented in Figure 1. 20,248 pregnancies (regardless of outcome) were eligible for enrolment which corresponds to 19,637 unique G0 mothers (611 mothers having two eligible pregnancies). During the initial core phase of recruitment, 14,541 pregnancies were enrolled, from 14,203 unique G0 mothers (338 mothers having two enrolled pregnancies).

630 mothers who did not enrol during pregnancy but have provided data since the G1 child was seven years of age are denoted as the non-core enrolled sample. It should be noted that 906 additional pregnancies, resulting in 913 G1 offspring, contribute to this non-core recruitment phase – these are described in the update to the G1 cohort profile elsewhere ^4^. There are 211 mothers who did not formally enrol in ALSPAC themselves, although their pregnancy was assumed to be enrolled because the associated G1-offspring is enrolled (the majority of these enrolments occurred after the G1 child was 18 years of age, and hence are non-core G1 offspring enrolments).

In total, therefore, there are 14,833 mothers enrolled in ALSPAC as of September 2021, with 15,447 associated pregnancies enrolled.

### G0 mothers with two or more enrolled pregnancies

In the enrolled sample, four mothers had three eligible pregnancies – in each case one of these pregnancies was either never formally enrolled or, if enrolled, had no data associated with it. For reasons of confidentiality, these have been edited so that each mother is only linked to two pregnancies.

There are 403 (2.7% of total sample) G0 mothers who had two pregnancies enrolled. Of these, 338 (83.9%) mothers had both pregnancies enrolled in the core ALSPAC sample, 57 (14.1%) had one pregnancy core enrolled and one non-core enrolled, while 8 (2.0%) had both pregnancies enrolled in the non-core ALSPAC sample. The outcomes for these pregnancies are summarised in Table 1. The majority of G0 mothers with two enrolled pregnancies had two singleton pregnancies (96.8%), with the remainder consisting of one singleton and one twin pregnancy. For approximately 50% of these mothers, only one pregnancy resulted in a live birth Table 1.

**Table 1.** Pregnancy outcomes for the 403 G0 mothers with two pregnancies in the enrolled ALSPAC cohort and for the 338 G0 mothers where two pregnancies are enrolled in the core ALSPAC sample.

Pregnancy outcome (variable name in MZ file)	Enrolled sample ( *n* = 403)	Core enrolled sample ( *n* = 338)
**Pregnancy size ( *mz010*)**		
Both pregnancies single births	390 (96.8%)	328 (97.0%)
One singleton and one twin birth (or one singleton birth and missing data for other pregnancy)	13 (3.2%)	10 (3.0%)
**Live birth ( *mz012*)**		
Live birth in both pregnancies	203 (50.4%)	155 (45.9%)
Live birth in only one pregnancy	191 (47.4%)	174 (51.5%)
No live births in either pregnancy	9 (2.2%)	9 (2.7%)
**Alive at 1 year of age ( *mz014*)**		
Child(ren) alive at 1 year in both pregnancies ^a^	189 (46.9%)	143 (42.3%)
Child(ren) alive at 1 year in only one pregnancy	205 (50.9%)	186 (55.0%)
No child(ren) alive at 1 year in either pregnancy	9 (2.2%)	9 (2.7%)

^a^ Note that this includes a twin birth where only one twin survived to one year.

It is important to note that the number of G0 mothers with two core enrolled pregnancies described here differs from that in the previous ALSPAC-G0 mothers cohort profile description ^1^. In that publication, we reported that after core enrolled pregnancies with unknown outcomes and non-live births were removed, there were 13,867 pregnancies associated with 13,761 unique mothers, meaning 106 mothers with multiple enrolled pregnancies (see Figure 1 of 1). However, using the most recent data, we can now report that of those 13,867 pregnancies as there were 13,712 unique mothers, of whom 155 mothers had multiple enrolled pregnancies. This discrepancy is likely a result of the previous publication using older data files which did not link together all pregnancies from the same mother; as the current publication uses the most recent records, the numbers reported here should be used going forwards.

### Guidance for analysing datasets including mothers with two enrolled pregnancies

**Figure 2.**
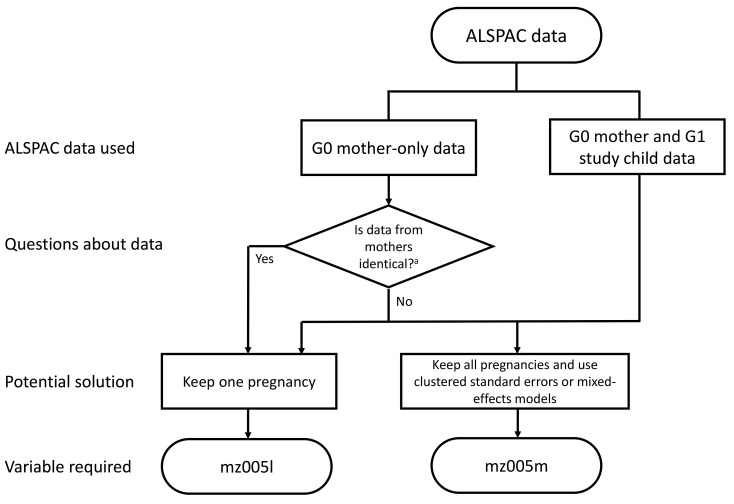
A flow diagram detailing potential methods to account for non-independence of data points, and which ‘mz’ variables to employ, when using ALSPAC data to account for the inclusion of mothers with two enrolled pregnancies. ^a^ In questionnaires ‘A’ through ‘U’ (plus ‘XA’, ‘XB’ and ‘W’) mothers completed separate questionnaires for each pregnancy, so the data may not be identical (depending on the question). In the ‘V’ and ‘Y’ and G0 mother COVID-19 questionnaires, plus all of the ‘Focus on Mothers’ clinics and associated biosamples data from these clinics, the mother’s data was only collected once, so is identical. However, we caution that this split into ‘identical’ vs ‘non-identical’ G0 mothers’ data may not apply in all cases and urge researchers to inspect their data carefully to see whether data from both pregnancies are identical or not.

Commonly used statistical tests and models assume independence ^5, 6^. For some ALSPAC data collection events the focus has been on the G0 mother, meaning that data linked to both pregnancies will be identical (e.g., the ‘Focus on Mother’ clinics, or mother-completed questionnaires which are specifically about the mother, rather than a particular pregnancy or child). At other times, the G0 mother’s data may not be identical, such as a questionnaire that has been completed twice, for each of the individual pregnancies. Although the data may not be identical, it is still likely to be non-independent, as a mother’s state at time *t* is likely to correlated with the mother’s state at time *t*+1. Variables have therefore been created to assist researchers working with these cases and a flow chart designed to guide decision making ( Figure 2). This is necessary because the unique identifier in ALSPAC is a pregnancy identifier, not a G0 mother identifier, meaning the pregnancy identifier cannot say whether pregnancies are from the same G0 mother. However, we note that due to the relatively small numbers of mothers with multiple pregnancies any potential bias or spurious precision due to this non-independence is likely to be small, and stress that it is up to individual researchers how they use and analyse ALSPAC data relative to their research question(s). The methods below are therefore suggestions that researchers may want to consider when using this data.

### G0 mother data linked to G1 child data

When working with G0 mother data linked to G1 child data, researchers may wish to consider using either robust standard errors clustered on G0 mother ID ^7^ or mixed-effects models with G0 mother ID as a random effect ^5^. The variable *mz005m* has been created which uniquely defines the mothers with two pregnancies in the eligible ALSPAC sample. Researchers could populate the missing values in this variable (i.e., mothers with only one enrolled pregnancy) with their pregnancy-specific ALSPAC collaborator ID; this variable could then be used as a random effect in a mixed-effects model, or as a cluster factor to create robust standard errors, to account for possible bias/spurious precision and/or explore possible between-pregnancy heterogeneity. Alternatively, given the small number of mothers with two pregnancies, researchers may simply choose to randomly drop one of the pregnancies, or select just the first or second pregnancy.

### G0 mother data only

When working with G0 mother data only (i.e., not linked to G1 data), potential methods for dealing with multiple pregnancies will vary depending on how the data were collected. In early mother-completed questionnaires, data were predominantly pregnancy-based, or completed at a particular time related to the age of each study child. Each mother may therefore have completed a separate questionnaire for each pregnancy; as such, the data may not be identical on each occasion, but are potentially correlated. In these cases, researchers may wish to use the procedure described above (i.e., using clustered standard errors, mixed-effects models, or simply removing one pregnancy).

In later data collections the focus has been on the mothers. Therefore, mothers completed one questionnaire or attended a single clinic visit. If researchers are using these data, we recommend they simply remove data associated with one of the mother’s pregnancies.

**Figure 3.**
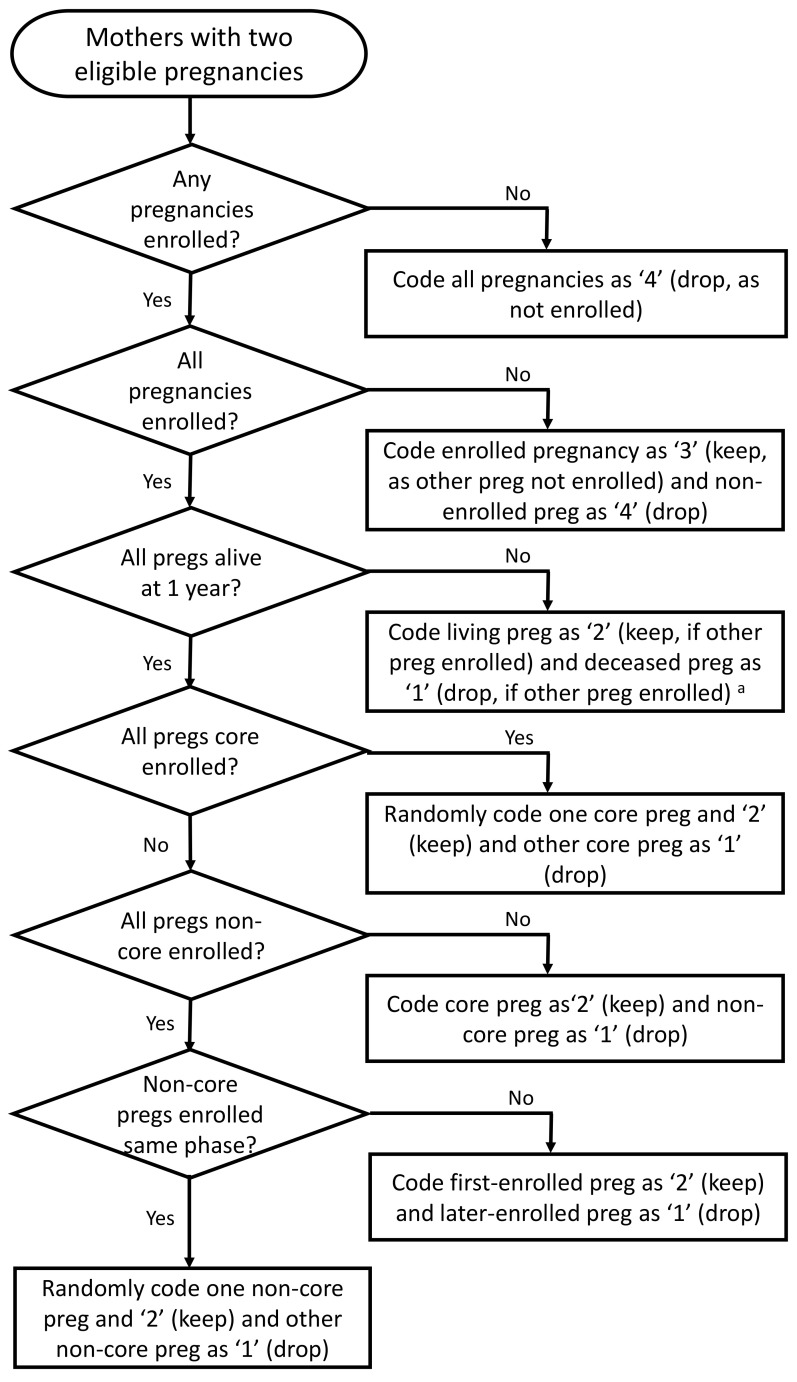
A flow diagram detailing how each of the 1,222 pregnancies from 611 mothers with two eligible pregnancies were coded as ‘keep’ or ‘drop’ in variable *mz005k* in Table 2. ^a^ Note that there are nine mothers with two pregnancies where the G1 children from both core pregnancies were not alive at 1 year of age. For these pregnancies, one pregnancy was randomly coded as ‘2’ (keep’) and the other as ‘1’ (‘drop’).

To assist researchers in doing so, we created the *mz005l* variable indicating which pregnancies to keep and which to drop, in order to reduce the dataset to one observation per mother ( Table 2). The 1,222 pregnancies from mothers with two eligible pregnancies were coded into one of four categories (summarised in variable *mz005k*; see Table 2). Decisions were hierarchical, regarding whether the pregnancy was enrolled in ALSPAC, whether the child from the pregnancy was alive at 1 year of age or not, and the phase of G1 study child recruitment (see decision process in Figure 3). To create variable *mz005l*, both ‘keep’ categories of variable *mz005k* (values of ‘2’ or ‘3’), plus all other mothers with only one pregnancy enrolled in ALSPAC, were combined into the ‘2’ category (meaning ‘keep all these cases’). The other two ‘drop’ categories of variable *mz005k* were combined in variable *mz005l*, to indicate that these pregnancies should be dropped.

**Table 2.** Descriptive statistics for variables relating to G0 mothers with two pregnancies in ALSPAC, and the decision on whether to ‘keep’ or ‘drop’ each pregnancy. Note that in variable *mz005* *l* all mothers with only one enrolled pregnancy are coded as ‘2’ (‘keep all these cases’). To assist researchers, variable *mz005l* will be provided with all data requests by default.

Description (variable name)	Count
**Observations to drop or keep just one pregnancy per mother ( *mz005l*)**	***n* = 20,248**
1: Yes, drop these pregnancies	673 (3.3%)
2: No, keep these pregnancies	19,575 (96.7%)
**Details of keep vs drop decisions for mothers with two pregnancies ( *mz005k*)**	***n* = 1,222**
1: Pregnancy to drop (if data from other pregnancy is enrolled in ALSPAC)	403 (33.0%)
2: Pregnancy to keep (if data from other pregnancy is enrolled in ALSPAC)	403 (33.0%)
3: Pregnancy to keep (if other pregnancy not enrolled in ALSPAC)	146 (11.9%)
4: Pregnancy to drop (as pregnancy not enrolled in ALSPAC)	270 (22.1%)

In variable *mz005l*, a value of ‘1’ therefore indicates a G0 mother with two eligible pregnancies and this is the pregnancy to drop; this could be because both pregnancies are enrolled and this is the pregnancy to drop, or the pregnancy is not enrolled in ALSPAC and so should also be removed as well. A value of ‘2’ in variable *mz005l* denotes a G0 mother only linked to one pregnancy, a mother with two enrolled pregnancies and this is the pregnancy to keep, or a mother with two eligible pregnancies where the other pregnancy is not enrolled in ALSPAC; regardless of the reason, all of the pregnancies meeting any of these criteria should be retained. Note when using these variables to remove duplicate mothers, there are 19 pregnancies where the mother is enrolled in the core ALSPAC sample but the pregnancy to keep is part of the non-core sample; this occurs because these 19 mothers have two pregnancies enrolled in ALSPAC; one in core which did not survive to 1 year (hence why the mother is core enrolled), while the other is a non-core enrolled pregnancy, which is the one to keep.

### Known G0 mother mortality

Information about deaths in the G0 mothers has been added to the *mz* file and was taken from ALSPAC’s internal administrative records. In the early years of the Study this data was obtained directly from official records, but subsequently relied upon the Study being informed of a death (e.g., by a relative). As such, there are likely to be deaths that are not currently recorded in ALSPAC’s administrative records, such as deaths occurring after ALSPAC had lost contact with the mother, or deaths occurring without ALSPAC being directly informed. Of the 14,833 G0 mothers enrolled in ALSPAC, 357 (2.4%) are known to have died prior to November 2021 (variable *mz100a*). The grouped dates of these known deaths are provided in variable *mz100b* with 20 (5.6%) of deaths between 1991-1999, 58 (16.2%) between 2000-2004, 79 (22.1%) between 2005-2009, 119 (33.3%) between 2010-2014 and 81 (22.7%) in 2015 or later. These numbers are almost certainly underestimates, particularly the later data – medical record linkage work using data from the Office for National Statistics is currently ongoing and will provide more accurate data in the future. Going forwards, the death variables will be updated on a regular basis to provide up-to-date mortality data.

## Summary

This data note has provided an update to the *mz* file which details ALSPAC-G0 mothers enrolment status. In total, 14,833 G0 mothers are enrolled, with 15,447 enrolled pregnancies.

This data note should be useful for all researchers working with the G0 mother ALSPAC data to understand the G0 mother enrolment methods and sample sizes, details of mothers with two pregnancies, and an update on G0 mother mortality.

## *mz* Cohort Profile Data File

The current *mz* mother’s cohort data file is *mz_6a*. Variable *mz005l* listed in Table 2, in addition to the variable which identifies mothers with two pregnancies ( *mz005m*), will be provided by default with all data requests. This file includes all 20,248 eligible pregnancies, although note that some of the enrolled G0 mothers and G1 study children have subsequently formally withdrawn from the study or are at high risk of disclosure (e.g., triplet or quadruplet pregnancies); these cases are included here, but their data is suppressed when accessing ALSPAC data.

Note that technically the *mz* file is based on the pregnancy cohort, rather than the mother’s cohort. This is why for some variables the focus is on the mother, while for others the focus is on the pregnancy. This complication is an inevitable consequence of the ALSPAC study design where mothers with multiple eligible pregnancies were possible; we have endeavoured to make this as clear as possible for users.

## Implications for ALSPAC data users

The previous G0 mother’s release files for data collections where data from mothers with two pregnancies were duplicated (‘V’, ‘Y’ and G0 mother COVID-19 questionnaires, plus all of the ‘Focus on Mothers’ clinics and associated biosamples data from these clinics) had a marker variable to indicate which observations to keep vs drop. These have now been removed and instead centralised in the *mz* file, as described above. The variables *mz005l* and *mz005m* will now be given out as standard with all ALSPAC data files in order for these multiple pregnancies to be identifiable.

Note that in subsequent G0 mother release files, and in datasets created for research purposes using **only** G0 data, the total sample size will be 15,326 (see variable *mum_and_preg_enrolled* in the *mz* file). This consists of all pregnancies where both the G0 mother and the pregnancy (or G1 child) are enrolled in ALSPAC. To reduce the dataset to just the 14,833 unique G0 mothers enrolled in ALSPAC, simply select all observations where variable *mz005l* is coded as ‘Keep’ (value of ‘2’). If this data is being matched to G1 data, the appropriate sample size will be provided for the number of children in the study (see 2 and 4).

If you have any questions about the data, or how to access it, please email alspac-data@bristol.ac.uk.

## Consent

Ethical approval for this study was obtained from the ALSPAC Ethics and Law Committee and the Local Research Ethics Committees. Informed consent for the use of data collected via questionnaires and clinics was obtained from participants following the recommendations of the ALSPAC Ethics and Law Committee at the time. Study participants have the right to withdraw their consent for elements of the study or from the study entirely. Full details of the ALSPAC consent procedures are available on the study website.

## Data Availability

ALSPAC data access is through a system of managed open access. The steps below highlight how to apply for access to the data included in the data note and all other ALSPAC data: Please read the ALSPAC access policy ( http://www.bristol.ac.uk/media-library/sites/alspac/documents/researchers/data-access/ALSPAC_Access_Policy.pdf) which describes the process of accessing the data and samples in detail, and outlines the costs associated with doing so. You may also find it useful to browse our fully searchable research proposals database ( https://proposals.epi.bristol.ac.uk/?q=proposalSummaries), which lists all research projects that have been approved since April 2011. Please submit your research proposal ( https://proposals.epi.bristol.ac.uk/) for consideration by the ALSPAC Executive Committee. You will receive a response within 10 working days to advise you whether your proposal has been approved.
