# Supplementary material for: The Avon Longitudinal Study of Parents and Children (ALSPAC): a 2022 update on the enrolled sample of mothers and the associated baseline data
Source: Wellcome Open Res. 2023 Sep 6;7:283. Originally published 2022 Nov 18. [Version 2] doi: 10.12688/wellcomeopenres.18564.2 (PMC10472060; doi:10.12688/wellcomeopenres.18564.2)
Supplement: Peer review history

# Peer review: Reviewer response for version 1

**DOI:** 10.21956/wellcomeopenres.20584.r64259

**Published:** 2023-08-31

**Authors:** Andrew O Agbaje

**Affiliations:** 1 Institute of Public Health and Clinical Nutrition, Faculty of Health Sciences, University of Eastern Finland, Kuopio, Finland; 2 Children's Health and Exercise Research Centre, Department of Public Health and Sports Sciences, Faculty of Health and Life Sciences, University of Exeter, Exeter, England, UK

This is an important update of the ALSPAC cohort, especially the information of the enrolled core and non-core mothers. Just some minor comments to consider.

 Kindly clarify whether these mothers with previously assumed enrollment were among the core or non-core mothers - "There are 211 mothers who did not formally enrol in ALSPAC themselves, although their pregnancy was assumed to be enrolled…"

 Please reference Table 1 which clarifies this statement, and prevents wrong interpretation such as linking the statement to the entire G0 mothers: "For approximately 50% of mothers, only one pregnancy resulted in a live birth..."

 A minor typo error in the sentence, kindly correct: " **The** reduce the dataset to just the 14,833 unique G0 mothers enrolled in ALSPAC, simply select all observations where variable mz005l is coded as ‘Keep’ (value of ‘2’)."

Are sufficient details of methods and materials provided to allow replication by others?

Yes

Is the rationale for creating the dataset(s) clearly described?

Yes

Are the datasets clearly presented in a useable and accessible format?

Yes

Are the protocols appropriate and is the work technically sound?

Yes

Reviewer Expertise:

Preventive cardiology, endocrinology, exercise medicine, epidemiology, and public health, in the paediatrics and young adult population.

I confirm that I have read this submission and believe that I have an appropriate level of expertise to confirm that it is of an acceptable scientific standard.

# Peer review: Reviewer response for version 1

**DOI:** 10.21956/wellcomeopenres.20584.r59440

**Published:** 2023-08-03

**Authors:** Delyse M Hutchinson

**Affiliations:** 1 Centre for Social and Early Emotional Development, School of Psychology, Deakin University, Geelong, Australia; 2 Department of Paediatrics, The University of Melbourne, Melbourne, Victoria, Australia; 3 National Drug and Alcohol Research Centre, University of New South Wales, Sydney, New South Wales, Australia; 4 Centre for Adolescent Health, Murdoch Childrens Research Institute, Melbourne, VIC, Australia

﻿This paper aims to provide an update on the current ALSPAC mother sample, including up to date mortality data, accounting for additional participating mothers since the last cohort profile was published. Secondly, the paper aims to provide guidance on how to manage non-independence between data points. 

 This represents an important paper for potential data users to identify women correctly in the sample, their pregnancies/offspring, and to account for non-independence between data points. 

 I recommend that this paper be indexed given its practical importance for understanding and appropriately analysing ALSPAC data.

 I do note that in the abstract it would aid the reader to provide definitions of new terms (e.g., G1 and G0). 

 In the introduction a brief explanation could be provided for the the ALSPAC@30 clinic (e.g., follow-up of G1 offspring at age 30).

 There are also a number of minor typos to be addressed during the final editorial process.

 Whilst not the focus of this paper, it would be helpful in a future publication to understand whether the characteristics of core and non-core participating mothers differ, and what the implications of including these additional mothers may be for data interpretation and generalisability.

Are sufficient details of methods and materials provided to allow replication by others?

Yes

Is the rationale for creating the dataset(s) clearly described?

Yes

Are the datasets clearly presented in a useable and accessible format?

Yes

Are the protocols appropriate and is the work technically sound?

Yes

Reviewer Expertise:

Mental health, substance use, perinatal, child development

I confirm that I have read this submission and believe that I have an appropriate level of expertise to confirm that it is of an acceptable scientific standard.
